# Sacubitril/valsartan as a modulator of pulmonary fibrosis: insights into Lnc-SNHG-16/miR-455 modulation and Notch/Smad-3 pathway inhibition

**DOI:** 10.1007/s00210-026-05034-0

**Published:** 2026-02-18

**Authors:** Zeinab M. Abdel-Nasser, Mai A. Zaafan

**Affiliations:** 1 Biochemistry Department, Faculty of Pharmacy, October University for Modern Sciences and Arts (MSA), Giza, Egypt; 2 Pharmacology & Toxicology Department, Faculty of Pharmacy, October University for Modern Sciences and Arts (MSA), Giza, Egypt

**Keywords:** Pulmonary fibrosis, Sacubitril/valsartan, TGF-β, Notch-2/Smad-3, SNHG-16/miR-455

## Abstract

Pulmonary fibrosis is a progressive and life-threatening interstitial lung disease marked by excessive extracellular matrix accumulation and declining pulmonary function. Despite available therapies, clinical outcomes remain suboptimal, necessitating the exploration of novel therapeutic approaches. Sacubitril/valsartan, a dual-acting agent combining a neprilysin inhibitor and angiotensin II receptor blocker, has recently emerged as a candidate with potential anti-fibrotic properties. This study investigates the therapeutic efficacy of sacubitril/valsartan in a bleomycin-induced rat model of pulmonary fibrosis. The compound was administered orally once daily, beginning on the first day of fibrosis induction and continued for 21 days. Fibrotic progression was evaluated through biochemical and histological assessments, including quantification of hydroxyproline content, α-smooth muscle actin (α-SMA) expression, collagen deposition percentage, and histopathological examination of lung tissue. Molecular analyses revealed that sacubitril/valsartan significantly downregulated the expression of long non-coding RNA SNHG-16 and concurrently upregulated miR-455 levels. These changes were associated with suppression of the Notch-2/Smad-3 signaling pathway. Additionally, sacubitril/valsartan treatment resulted in a notable reduction in pulmonary levels of transforming growth factor-β (TGF-β), interleukin-6 (IL-6), and tumor necrosis factor-α (TNF-α), indicating attenuation of both fibrotic and inflammatory responses. Collectively, these findings suggest that sacubitril/valsartan mitigates pulmonary fibrosis through modulation of the SNHG-16/miR-455 axis and inhibition of the Notch-2/Smad-3/TGF-β signaling cascade, highlighting its potential as a promising therapeutic strategy for the management of pulmonary fibrosis.

**Graphical Abstract:**

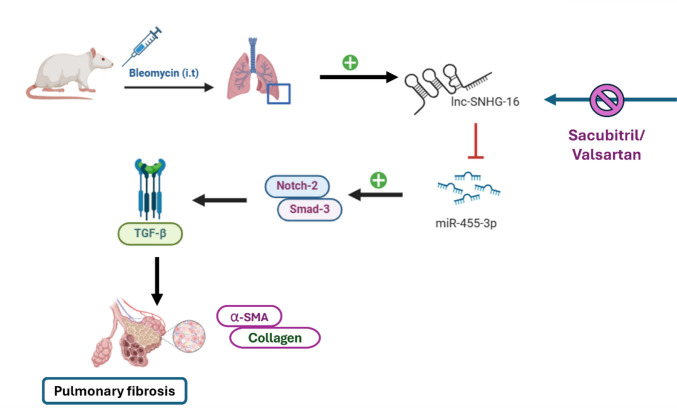

## Introduction

Pulmonary fibrosis (PF) is a progressive interstitial lung disease characterized by aberrant accumulation of extracellular matrix (ECM) components, leading to increased tissue rigidity, disruption of normal lung architecture, and irreversible scarring of the pulmonary parenchyma. These pathological alterations compromise alveolar gas exchange, ultimately resulting in chronic respiratory insufficiency and, in advanced stages, fatal respiratory failure. Idiopathic pulmonary fibrosis (IPF) is the most common and clinically severe form of PF, with a median survival ranging from 2 to 6 years following diagnosis, despite the availability of current therapeutic modalities (El-Kashef et al. 2022). Epidemiological data indicate a notable rise in IPF incidence since the early 2000 s (Strongman et al. 2018), with a disproportionately higher prevalence observed among elderly individuals, particularly those aged over 60 years (Koudstaal And Wijsenbeek 2023).

The pathogenesis of pulmonary fibrosis is multifactorial, involving a complex interplay of cytokine release, recruitment and activation of diverse cellular populations, and dysregulation of multiple intracellular signaling pathways (Strieter And Mehrad 2009). Emerging evidence implicates aberrations in the Notch signaling pathway, chronic inflammation, and impaired autophagy as key contributors to disease progression (Shi et al. 2024). Within the context of PF, Notch signaling has been shown to promote epithelial–mesenchymal transition (EMT) in alveolar epithelial cells via transforming growth factor β1 (TGF-β1), thereby initiating interstitial fibrosis and enhancing α-smooth muscle actin (α-SMA) expression, a hallmark of myofibroblast differentiation (Abd El-Hameed et al. 2025; Chen et al. 2020). Increasing attention has been directed toward the regulatory roles of non-coding RNAs, particularly microRNAs (miRNAs) and long non-coding RNAs (lncRNAs), in modulating gene expression and cellular processes associated with fibrogenesis (Abdel-Nasser et al. 2023; Zaafan and Abdelhamid 2025). The lncRNA Small Nucleolar RNA Host Gene 16 (SNHG-16) has been identified as a pivotal mediator in various pathological conditions, including fibrosis (Ghafouri-Fard et al. 2021). SNHG-16 is proposed to function as a competing endogenous RNA (ceRNA), sequestering specific miRNAs and thereby influencing the expression of downstream targets. In PF, SNHG-16 is hypothesized to interact with microRNA-455 (miR-455), a miRNA implicated in the regulation of fibrotic pathways. MiR-455 has been shown to target and suppress Notch2, a critical component of the Notch signaling cascade involved in cellular differentiation, proliferation, and apoptosis (Ayeldeen et al. 2024; Abdelhamid et al. 2022). Dysregulation of Notch2 has been directly associated with PF pathogenesis (Liu et al. 2021b). Growing evidence underscores that modulating the Notch signaling pathway is critical for attenuating the molecular drivers of myofibroblast activation and subsequent tissue scarring. Furthermore, restoring cellular homeostasis through the dual suppression of chronic inflammation and the reversal of impaired autophagy represents a comprehensive approach to halting the progression of chronic interstitial lung diseases (Fadaly et al. 2024).

Sacubitril/valsartan (Sac/Val) is a first-in-class angiotensin receptor-neprilysin inhibitor (ARNI). Sacubitril acts as a prodrug that is metabolized into the active neprilysin inhibitor. By inhibiting neprilysin, it prevents the degradation of natriuretic peptides, thereby enhancing vasodilation and natriuresis. Conversely, valsartan provides a potent, selective blockade of the Angiotensin II Type 1 (AT-1) receptor, preventing the pro-fibrotic and vasoconstrictive effects of the renin–angiotensin–aldosterone system (RAAS). This dual mechanism is hypothesized to provide a superior synergistic effect compared to traditional ACE inhibitors or ARBs alone in remodeling-dependent diseases (Brioschi et al. 2023). Sac/Val has demonstrated the ability to inhibit the TGF-β1/Smad3 signaling pathway during cardiac fibrosis progression (Zhang et al. 2020). Additionally, Sac/Val has been reported to attenuate cardiac fibrosis and improve cardiac function by reducing fibroblast proliferation (Burke et al. 2019). Accordingly, the present study aims to evaluate the therapeutic potential of sacubitril/valsartan in a bleomycin-induced rat model of PF. We hypothesize that the dual inhibition of neprilysin and the AT-1 receptor will attenuate pulmonary fibrogenesis by suppressing inflammatory cytokines and reversing extracellular matrix deposition. Crucially, we aim to elucidate the molecular involvement of the SNHG-16/miR-455-3p/Notch-2/Smad-3 signaling cascade, providing a mechanistic basis for the use of ARNIs as a novel strategy for the management of progressive lung fibrosis.

## Materials and methods

### Animals

Male Wistar albino rats, weighing between 150 and 200 g, were obtained from the Teodor Bilharz Research Institute (Cairo, Egypt) for use in this study. Animals were housed in standard plastic cages under controlled environmental conditions (temperature, 25 ± 3 °C; relative humidity, 50%) in the animal facility of MSA University. Rats were maintained on a standard pellet diet and provided with unrestricted access to water throughout the experimental period.

### Induction of pulmonary fibrosis

A single intratracheal bleomycin sulphate injection (5 mg/kg) was used for the experimental induction of pulmonary fibrosis in rats. Anesthesia was induced using a ketamine/xylazine combination (80/20 mg/kg, i.p.) (Zaafan et al. 2019).

### Experimental design

A total of 18 rats were randomly assigned to three groups (*n* = 6 per group) using the standard = RAND() function in Microsoft Excel. The sample size was calculated using G*Power software based on anticipated differences in pulmonary hydroxyproline levels, with a statistical power of 80%. The first group was the normal group, and the second group (PF-control group) was injected with bleomycin (5 mg/kg; i.t.). The third group received sacubitril/valsartan for 21 days starting on the day of induction (68 mg/kg, p.o.) (An et al. 2022). Finally, the animals were sacrificed by cervical dislocation. The lungs were rapidly isolated and used for biochemical investigations and histological analysis.

### Biochemical assays

The hydroxyproline content was investigated in lung tissue using a standard colorimetric assay kit (CAT no. K555-100; Biovision incorp, CA, USA). The pulmonary contents of tumor necrosis factor-α (TNF-α, cat. no. 438204, BioLegend, CA, USA), interleukin-6 (IL-6; cat. no. SEA079Ra, Cloud Clone Carp., TX, USA), and α-smooth muscle actin (α-SMA; cat. no. NBP2-66,430, Novus Biologicals, CO, USA) were investigated with the ELISA technique using the following kits according to the directions of the manufacturer. Total RNA was extracted from lung tissue of all the experimental groups using direct-zol RNA Miniprep Plus (Cat. no. R2072, ZYMO RESEARCH CORP. USA). After that, a Beckman dual spectrophotometer (USA) was used for quantity and quality assessment.

Reverse transcription and RNA extraction were performed using SuperScript IV One-Step RT-PCR kit (Cat# 12,594,100, Thermo Fisher Scientific, Waltham, MA, USA) followed by PCR using 96-well plate StepOne instrument (Applied Biosystem, USA). The data were presented as cycle threshold (Ct) values for both housekeeping and target genes. Normalization of the expression of target genes (Smad3, TGF-β, Notch2, and SNHG16) was performed relative to the mean critical threshold (Ct) value of the GAPDH housekeeping gene. For microRNA (miR-455-3p), the small nuclear RNA U6 was utilized as the internal reference. Relative expression levels were calculated using the 2^−∆∆Ct^ method.

The primer sequences used in the current study were as follows:

**Table:** 

Gene	Forward primer	Reverse primer
Smad-3	5′-GGCTTTGAGGCTGTCTACCA-3′	5′-GGTGCTGGTCACTGTCTGTC-3′
TGF-β	5′-GACTCTCCACCTGCAAGACC-3′	5′-GGACTGGCGAGCCTTAGTTT-3′
Notch2	5′-TTTGCTGTCGGAAGACGACC-3′	5′-GCCCATGTTGTCCTGGGCGT-3′
Lnc-RNA-SNHG16	5′-CAGAATGCCATGGTTTCCCC-3′	5′-TGGCAAGAGACTTCCTGAGG-3′
miR-455-3p	5'-CCCATCACCATCTTCCAGGAG-3'	5′-GCAGTCCATGGGCATATACAC-3′
GAPDH	5'-CCCATCACCATCTTCCAGGAG-3'	5'-GAAGGGGCGGAGATGATGAC-3'
U6	5′-ATGACGTCTGCCTTGGAGAAC-3′	5′-AGTGCAGGGTCCGAGGTATT-3′

The selection of assays for quantification was based on the specific biological characteristics and functional roles of each mediator. Notch2/Smad3/TGF-β1 were assessed via gene expression analysis (RT-qPCR) to evaluate the potential for de novo production and to better correlate with upstream signaling pathway activation where mRNA levels serve as a more direct indicator of cellular synthesis. In contrast, TNF-α and IL-6 were quantified at the protein level (ELISA) to provide a more accurate measure of their active, functional presence within the pulmonary tissue during acute inflammation. While protein assays offer high sensitivity for assessing immediate physiological effects, gene expression analysis was prioritized for investigating the long-term regulatory pathways involved in the fibrotic process.

### Histopathological examination of lung tissue

Lung tissues from different groups were washed and fixed in neutral formalin solution. After being trimmed and processed according to Culling (2013), tissue sections were cut with a rotatory microtome and stained with H&E stain as well as Masson’s trichome stain (Leica Microsystems GmbH, Wetzlar, Germany) and examined under a light microscope. For each tissue section in the sample, at least six representative, non-overlapping fields were randomly chosen and scanned to determine the area percentage of collagen fiber deposition in sections stained with Masson’s trichrome. The Full HD microscopic imaging system (Leica Microsystems GmbH, Germany) with Leica Application software for tissue section analysis was used to collect the data.

### Statistical analysis

All data were presented as mean ± S.E.M. The Shapiro–Wilk test was used to check the data normality. The Tukey–Kramer multiple comparisons test was used after the one-way ANOVA test to compare the means of the different groups. Following the Kruskel-Wallis test, the histopathological scores were assessed using Dunn’s multiple comparisons test. The significance threshold was established at *p* < 0.05. All statistical analyses were performed using GraphPad Prism version 8 (GraphPad Software, Inc., USA).

## Results

### Pulmonary contents of hydroxyproline and α-SMA

**Fig. 1:**
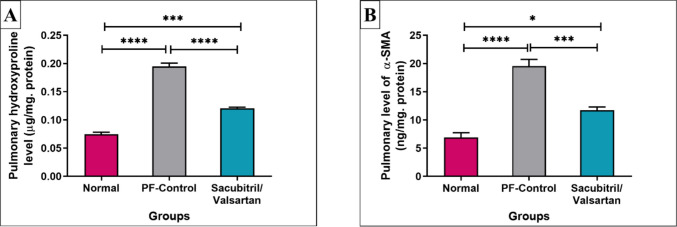
Effect of sacubitril/valsartan on pulmonary levels of (A) hydroxyproline and (B) α-smooth muscle actin (α-SMA). The data are expressed asmean ± SEM (sample no. per group = 6). Statistical analysis was performed using one-way ANOVA followed by the Tukey-Kramer multiple comparisons test. *, **, ***, ****: significant difference *p *˂ 0.05, 0.01, 0.001, and 0.0001, respectively

Results of the current study showed a significant increase in the pulmonary levels of hydroxyproline and α-SMA in the PF-control group compared to the normal group (*P* < 0.0001). On the contrary, pulmonary tissue from the sacubitril/valsartan-treated group showed a significant suppression of the levels of hydroxyproline (*P* < 0.0001) and α-SMA (*P* < 0.001) compared to the PF-control group (Fig. 1).

### Pulmonary expression levels of Lnc-SNGH-16 and miRNA-455-3p

**Fig. 2:**
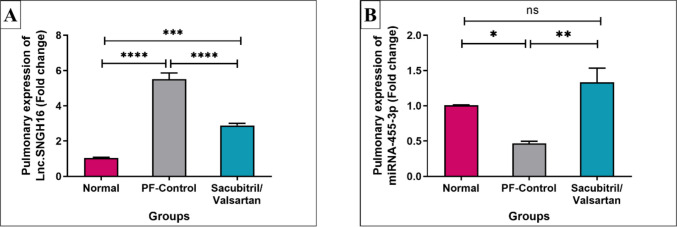
Effect of sacubitril/valsartan on the pulmonary expression levels of (A) Lnc-SNHH-16 and (B) miRNA-455. The data are expressed asmean ± SEM (sample no. per group = 6). Statistical analysis was performed using one-way ANOVA followed by the Tukey-Kramer multiple comparisons test.*, **, ***, ****: significant difference *p *< 0.05, 0.01, 0.001, and 0.0001, respectively

In the current study, pulmonary expression of Lnc-SNGH-16 was 4.4-fold higher in the PF-control group than in the normal group (*p* < 0.0001), a change accompanied by a significant suppression of miRNA-455-3p expression to 0.47-fold of that in the normal group (*p* < 0.05). Conversely, treatment with sacubitril/valsartan significantly attenuated these effects; pulmonary Lnc-SNGH-16 levels were reduced to approximately 0.53-fold of those in the PF-control group (*p* < 0.0001), while miRNA-455-3p expression levels significantly increased by 2.8-fold (*p* < 0.01) compared to the PF-control group (Fig. 2A and B).

### Pulmonary expression levels of Notch-2, Smad-3 and TGF-β

**Fig. 3:**
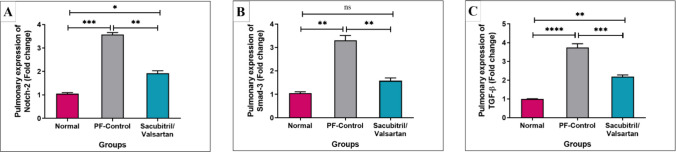
Effect of sacubitril/valsartan on the pulmonary expression levels of (A) Notch-2, (B) Smad-3 and (C) TGF-β. The data are expressed asmean ± SEM (sample no. per group = 6). Statistical analysis was performed using one-way ANOVA followed by the Tukey-Kramer multiple comparisons test.*, **, ***, ****: significant difference *p *˂ 0.05, 0.01, 0.001, and 0.0001, respectively

The current results revealed a significant increase in the pulmonary expression levels of Notch-2, Smad-3, and TGF-β in the PF-control group by 2.3-, 2-, and 2.8-fold, respectively, compared to the normal group (*P* < 0.001, 0.01, and 0.0001, respectively). Pulmonary expression levels of Notch-2, Smad-3, and TGF-β were significantly reduced upon treatment with sacubitril/valsartan by 0.47 (*p* < 0.01), 0.52 (*p* < 0.01), and 0.42-fold (*p* < 0.001), respectively, compared to the PF-control group (Fig. 3A, B, and C).

### Pulmonary contents of TNF-α and IL-6

**Fig. 4:**
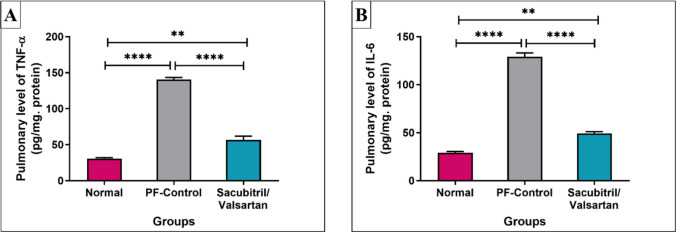
Effect of sacubitril/valsartan on the pulmonary (A) TNF-α and (B) IL-6 contents. The data are expressed asmean ± SEM (sample no. per group = 6). Statistical analysis was performed using one-way ANOVA followed by the Tukey-Kramer multiple comparisons test. *, **, ***, ****: significant difference *p *˂ 0.05, 0.01, 0.001, and 0.0001, respectively

Results of the current study showed a significant increase in the pulmonary TNF-α and IL-6 contents in the PF-control group compared to the normal group (*P* < 0.0001). On the contrary, pulmonary tissue from the sacubitril/valsartan-treated group showed a significant suppression of the levels of TNF-α and IL-6 (*P* < 0.0001) compared to the PF-control group (Fig. 4A and B).

### The histopathological examination of lung tissue

**Fig. 5:**
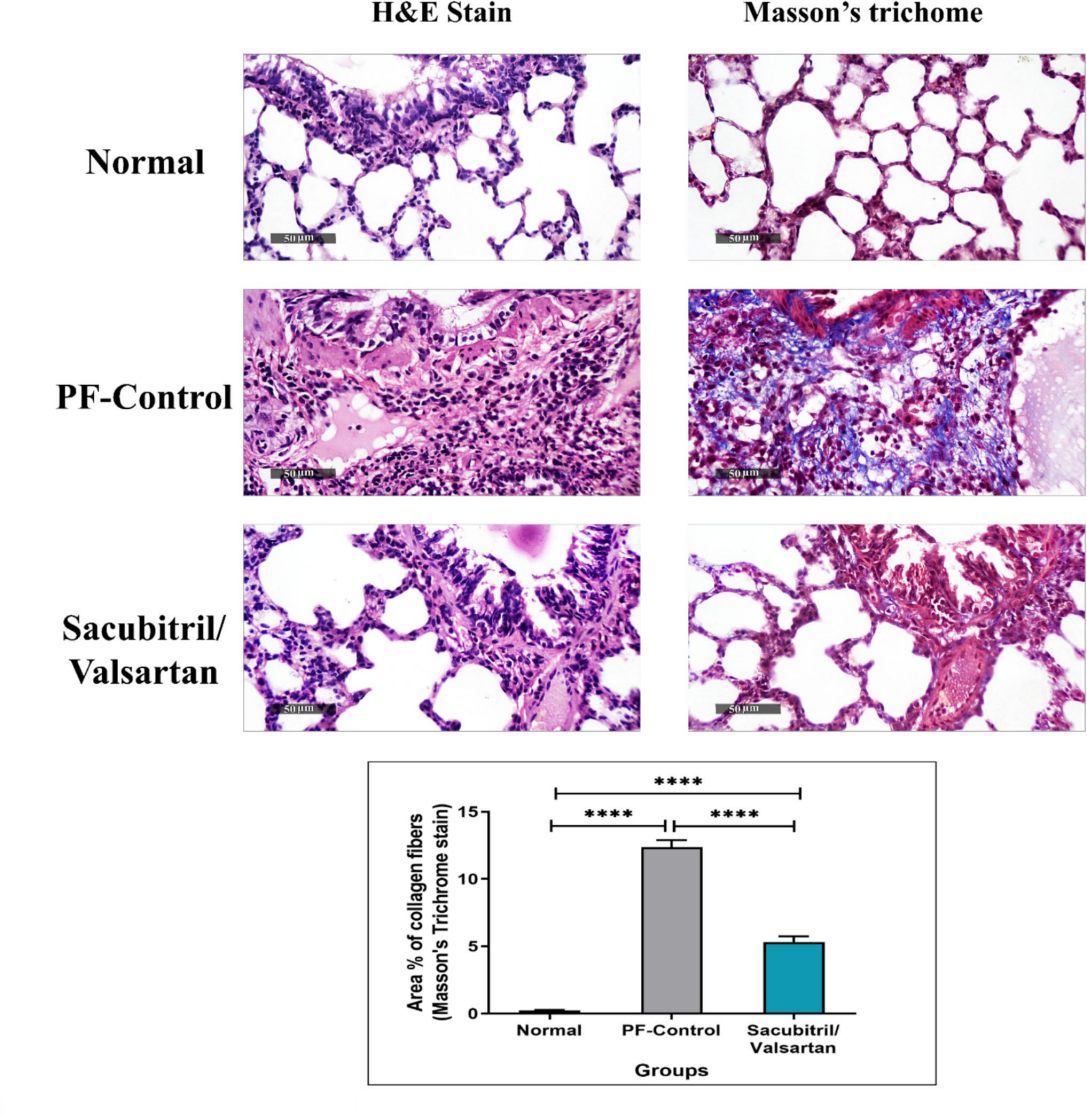
Histopathological examination of pulmonary tissue from all the different groups (using H&E stain as well as Masson’s trichome stain). Normal group: displays intact alveolar architecture and thin inter-alveolar walls with minimal inflammatory infiltration. PF-control: extensive peribronchiolar/intra-alveolar inflammation, and dense collagen deposition (blue staining) indicating significant fibroblastic changes. Sacubitril/valsartan treated group: exhibits marked pulmonary protection with reduced fibroblastic activity and only focal mild inflammatory infiltrates. Statistical analysis of the area percentage of collagen fibers showed that Sacubitril/valsartan treatment significantly suppressed collagen deposition compared with the PF-control group (****: significant difference, *p *< 0.0001)

Pulmonary tissue samples of the normal group showed intact histological features of the pulmonary parenchyma with an intact alveolar epithelium with thin interalveolar walls showing minimal inflammatory cell infiltrates and normal vasculatures. Pulmonary samples of the PF-control group demonstrate severe pneumonia with abundant peribronchiolar and intra-alveolar inflammatory cell infiltrates, as well as marked fibroblastic changes with higher collagen deposition in peribronchiolar spaces. Samples of the sacubitril/valsartan-treated group showed significant pulmonary protective efficacy and anti-fibroblastic activity, with focal mild periportal inflammatory cell infiltrates. Calculation of the area % of collagen fibers (Masson’s trichome stain) revealed a significant increase in collagen fiber deposition in the PF-control group compared with the normal group. On the other hand, treatment with sacubitril/valsartan resulted in a significant suppression of collagen fiber deposition and a marked improvement from the PF-control group (Fig. 5).

## Discussion

Pulmonary fibrosis is associated with a high mortality rate due to the progressive deterioration of respiratory function. Advancing our understanding of the underlying molecular mechanisms and identifying novel therapeutic targets are critical for the development of more effective treatment strategies. Sacubitril/valsartan, the first agent approved in the angiotensin receptor–neprilysin inhibitor (ARNI) class for the management of heart failure, combines the actions of sacubitril, a neprilysin inhibitor, and valsartan, an angiotensin II type 1 receptor (AT1R) antagonist. Neprilysin is a metalloprotease responsible for the degradation of circulating natriuretic peptides, which exert vasodilatory and antihypertensive effects. Consequently, sacubitril/valsartan exerts dual pharmacological actions by preserving natriuretic peptide levels and concurrently inhibiting the renin–angiotensin–aldosterone system (RAAS) (Cuthbert et al. 2020).

Beyond its cardiovascular indications, sacubitril/valsartan has demonstrated inhibitory effects on key signaling pathways implicated in cardiac fibrosis and extracellular matrix remodeling (Alhadad et al. 2025). Notably, it has been shown to exert cytoprotective effects through modulation of the TGF-β/Smad signaling axis (Mustafa et al. 2022; Abdelhamid et al. 2022). Consistent findings across various preclinical models of heart failure have revealed its capacity to suppress collagen synthesis and downregulate TGF-β1 and Smad3 expression, thereby attenuating fibrotic progression (Zaafan et al. 2016). These promising outcomes have laid the foundation for investigating the potential therapeutic efficacy of sacubitril/valsartan in pulmonary fibrosis, as explored in the current study.

The findings of the present study highlight a mechanistic interplay between long non-coding RNA SNHG-16, microRNA-455 (miR-455), and the Notch signaling pathway in the molecular pathogenesis of pulmonary fibrosis (PF). Pulmonary tissue analysis revealed a significant upregulation of SNHG-16 expression accompanied by a marked downregulation of miR-455-3p in the bleomycin-induced PF control group. This group also exhibited elevated levels of Notch-2 and Smad-3, supporting the hypothesis that SNHG-16 functions as a competing endogenous RNA (ceRNA), sequestering miR-455-3p and thereby promoting fibrogenesis through the depression of Notch-2 expression (Liu et al. 2021b). Activation of the Notch signaling pathway has been previously documented in patients with idiopathic pulmonary fibrosis and chronic obstructive pulmonary disease, as well as in experimental models of PF (Hu And Phan 2016). Enhanced Notch signaling contributes to fibrotic progression by stimulating fibroblast activation and myofibroblast differentiation, leading to excessive extracellular matrix deposition. Moreover, Notch signaling has been shown to upregulate TGF-β family members and induce Smad3 phosphorylation, both of which are central to fibrogenic signaling cascades (Elyaman et al. 2012; Ni et al. 2018). The strategic modulation of the Notch signaling pathway was reported to represent a pivotal therapeutic shift, as these interventions not only suppress chronic inflammation but also address a critical pathological hallmark of fibrosis by reversing impaired autophagy to restore cellular homeostasis (Abd El-Mawgoud et al. 2024).

Consistent with these molecular findings, the current investigation confirmed that the bleomycin model replicates the key histopathological features of PF, including fibrotic foci formation, inflammatory cell infiltration, and increased collagen content in the lung tissue. These pathological changes were further substantiated by elevated levels of α-smooth muscle actin (α-SMA) and hydroxyproline, along with increased concentrations of the pro-inflammatory cytokines TNF-α and IL-6. These results align with previous studies that have validated the bleomycin-induced rat model as a reliable representation of idiopathic pulmonary fibrosis (Abdelhady et al. 2023; Zaafan et al. 2019).

Treatment with sacubitril/valsartan resulted in a marked attenuation of fibrogenesis, as evidenced by histopathological examination of lung tissue, which revealed a significant reduction in collagen fiber deposition. This was further supported by a notable decrease in pulmonary levels of hydroxyproline and α-smooth muscle actin (α-SMA) compared with the bleomycin-induced PF control group. These findings are consistent with previous research demonstrating the anti-fibrotic potential of sacubitril/valsartan in a murine model of diabetic kidney disease, where it modulated the Sirt1/PGC1α pathway to maintain mitochondrial homeostasis and suppress tubulointerstitial fibrosis (Zhang et al. 2024).

In the present study, sacubitril/valsartan exerted its anti-fibrotic effects through modulation of the LncRNA SNHG-16/miR-455-3p/Notch signaling axis, accompanied by significant downregulation of Smad-3 and TGF-β, as well as a reduction in pro-inflammatory cytokines TNF-α and IL-6 within lung tissue. It was revealed that sacubitril/valsartan significantly downregulated the expression of long non-coding RNA (lncRNA) in the treated groups. This modulation likely stems from the ability of small-molecule drug components to engage in specific molecular interactions with RNA transcripts. Small molecules can recognize and bind to unique polynucleotide repeats or secondary structural motifs, thereby influencing RNA stability, processing, or translation (Nemr et al. 2019). In the context of sacubitril/valsartan, such interactions may interfere with the regulatory scaffolding provided by lncRNAs, leading to the observed downregulation and subsequent attenuation of downstream fibrotic signaling. Understanding these small-molecule-RNA interactions is crucial for elucidating the full pharmacological profile of combined therapies in complex disease states.

These findings align with previous reports of sacubitril/valsartan’s cardioprotective effects in models of cardiac fibrosis, where it was shown to regulate the TGF-β/Smad signaling pathway (Liu et al. 2021a). TGF-β1, a key mediator activated in response to cellular injury, initiates fibrotic signaling through receptor-mediated recruitment and activation of Smad2 and Smad3, ultimately leading to collagen deposition (Mustafa et al. 2022; Abdelhamid et al. 2021; Zaafan and Abdelhamid 2022).

Collectively, these results suggest that sacubitril/valsartan possesses promising anti-fibrotic potential in pulmonary fibrosis. While the anti-fibrotic properties of sacubitril/valsartan have been previously explored in renal and cardiac contexts, the originality of the current study lies in the identification of a specific epigenetic mechanism within the lung. This study demonstrates that sacubitril/valsartan can act as a molecular modulator of the LncRNA SNHG-16/miR-455-3p/Notch-2 signaling axis. Unlike traditional therapies that target inflammation broadly, our findings suggest that this ARNI combination provides a targeted “sponge” effect on miR-455-3p via SNHG-16 downregulation. This specific interaction offers a novel explanation for how small-molecule combined therapies can achieve high-precision control over the Notch signaling pathway, which is notoriously difficult to target without significant side effects. Finally, although we identified the SNHG-16/miR-455-3p/Notch-2 axis as a key target, our study relies on expression correlation. To definitively prove the necessity of this axis, future research utilizing silencing (siRNA) or mimics of miR-455-3p is required to confirm that the drug’s effects are strictly dependent on this epigenetic pathway.

## Conclusion

The current study demonstrates that sacubitril/valsartan exerts a significant protective effect against bleomycin-induced pulmonary fibrosis (PF). Our findings elucidate a multi-faceted therapeutic mechanism wherein the drug attenuates fibrotic progression by modulating the LncRNA SNHG-16/miR-455-3p/Notch-2 signaling axis. By acting as a molecular intervention that influences RNA stability and suppresses the Notch-2/Smad-3/TGF-β pathway, sacubitril/valsartan effectively reduces collagen deposition, myofibroblast differentiation (as evidenced by decreased α-SMA), and the secretion of pro-inflammatory cytokines such as TNF-α and IL-6. Furthermore, this research highlights the importance of dual-target pharmacological strategies to address the complex pathological hallmarks of PF, including the restoration of cellular homeostasis through the potential reversal of impaired autophagy. Collectively, these results suggest that sacubitril/valsartan, beyond its established cardiovascular applications, represents a promising candidate for drug repositioning in the management of fibroproliferative lung diseases.

## Data Availability

The datasets generated analyzed during the current study are available from the corresponding author upon reasonable request.
